# Optimizing BCPNN Learning Rule for Memory Access

**DOI:** 10.3389/fnins.2020.00878

**Published:** 2020-08-31

**Authors:** Yu Yang, Dimitrios Stathis, Rodolfo Jordão, Ahmed Hemani, Anders Lansner

**Affiliations:** ^1^Division of Electronics and Embedded Systems, School of Electrical Engineering and Computer Science, KTH Royal Institute of Technology, Stockholm, Sweden; ^2^Division of Computational Science and Technology, School of Electrical Engineering and Computer Science, KTH Royal Institute of Technology, Stockholm, Sweden; ^3^Department of Mathematics, Stockholm University, Stockholm, Sweden

**Keywords:** Bayesian Confidence Propagation Neural Network (BCPNN), neuromorphic computing, Hebbian learning, spiking neural networks, memory optimization, DRAM, cache, digital neuromorphic hardware

## Abstract

Simulation of large scale biologically plausible spiking neural networks, e.g., Bayesian Confidence Propagation Neural Network (BCPNN), usually requires high-performance supercomputers with dedicated accelerators, such as GPUs, FPGAs, or even Application-Specific Integrated Circuits (ASICs). Almost all of these computers are based on the von Neumann architecture that separates storage and computation. In all these solutions, memory access is the dominant cost even for highly customized computation and memory architecture, such as ASICs. In this paper, we propose an optimization technique that can make the BCPNN simulation memory access friendly by avoiding a dual-access pattern. The BCPNN synaptic traces and weights are organized as matrices accessed both row-wise and column-wise. Accessing data stored in DRAM with a dual-access pattern is extremely expensive. A post-synaptic history buffer and an approximation function thus are introduced to eliminate the troublesome column update. The error analysis combining theoretical analysis and experiments suggests that the probability of introducing intolerable errors by such optimization can be bounded to a very small number, which makes it almost negligible. Derivation and validation of such a bound is the core contribution of this paper. Experiments on a GPU platform shows that compared to the previously reported baseline simulation strategy, the proposed optimization technique reduces the storage requirement by 33%, the global memory access demand by more than 27% and DRAM access rate by more than 5%; the latency of updating synaptic traces decreases by roughly 50%. Compared with the other similar optimization technique reported in the literature, our method clearly shows considerably better results. Although the BCPNN is used as the targeted neural network model, the proposed optimization method can be applied to other artificial neural network models based on a Hebbian learning rule.

## 1. Introduction

Bayesian Confidence Propagation Neural Networks (BCPNNs), proposed by Lansner and Ekeberg (1989) and Lansner and Holst (1996), are biologically plausible brain cortex models that have been proven useful for understanding brain functions. Tully et al. (2016) implemented a BCPNN on SpiNNaker and analyzed the neural structure and dynamics inside a hypercolumn and demonstrated temporal sequence learning. Meli and Lansner (2013) studied the neural interconnection scheme from a BCPNN model. Fiebig et al. (2020) demonstrated how BCPNN could emulate the cortical working memory function. Recently, unsupervised hidden representation learning using BCPNN was benchmarked on MNIST. The BCPNN achieved 97.5% accuracy on the unseen test set (Ravichandran et al., 2020).

Currently, the simulation of large scale BCPNNs heavily relies on high-performance computing centers equipped with supercomputers and accelerators, such as GPUs and ASICs (Farahini et al., 2014; Stathis et al., 2020), or dedicated spiking neural network simulation platform, such as SpiNNaker (Knight et al., 2016). We identify three categories of optimization methods: (1) reducing the amount of computation, (2) reducing the amount of memory access demand, and (3) increasing the memory access efficiency. Current studies of the BCPNN optimization are mainly focused on reducing the computation and memory access demand (Vogginger et al., 2015). The memory access efficiency aspect is seldom exploited.

With technology scaling, memory access becomes the dominant cost for most applications (Mutlu, 2013). Memory optimization in terms of both reducing memory access demand and increasing the efficiency of the memory access has been done for many years both for non-Hebbian artificial neural networks and Hebbian spiking neural networks. For conventional non-spiking deep neural networks, research works like Li et al. (2016) and Yang et al. (2017) optimized both memory access demand and efficiency in deep convolutional neural networks. For Hebbian spiking neural networks, most research works target the spike-timing-dependent plasticity (STDP) learning rule (Markram et al., 2012). For example, Bichler et al. (2012) simplified the STDP learning rule and reduced the demand for computation and memory access. Yousefzadeh et al. (2017) further improved the method proposed by Bichler et al. by replacing the full connection to a weight-sharing connection. Thus, it further reduced the computation and memory access demand. Davies et al. (2012) changed part of the STDP learning rule and approximated the membrane potential in LTP. It reduced the computation and memory access demand and improved memory access efficiency. Jin et al. (2010) and Davies et al. (2018) delayed the update of weights and reduced the memory access demand. Pedroni et al. (2019) analyzed different synaptic matrix memory mapping strategies and proposed a variation of STDP learning rule to perform the causal and acausal update process. It reduced memory storage demand for the sparsely connected network by pointer-based compressed sparse rows and improved the efficiency for reversed access of such pointer-based data structure. Knight and Furber (2016) proposed a spike buffer and a mechanism called “flushing event” to deal with the inefficient column update in STDP and increase the memory efficiency. Morrison et al. (2007) used a dynamic spike buffer to remove column update process in STDP and increased memory efficiency. Sheik et al. (2016) also pointed out the memory problem caused by bi-directional spike-triggered learning rule and proposed a learning rule in which update is only triggered by presynaptic spikes to improve the memory access efficiency. However, almost all of these studies that target spiking neural networks focus on the STDP learning rule. Thus, it cannot be directly applied to BCPNN since its learning rule is different and more complex than STDP. By abandoning a conventional von Neumann architecture, custom neural network simulation platforms could potentially avoid the root of the memory access problems. Serrano-Gotarredona et al. (2013) and Prezioso et al. (2018) used memristors to merge computation and storage, thus eliminating the need for memory access. Though such new architectures are efficient and attractive, they are not off-the-shelf and easily accessible, and none of them supports the BCPNN learning rule.

In this paper, we tackle the memory access problem introduced by the BCPNN optimization method presented in Vogginger et al. (2015). By replacing a time-driven simulation method with an event-driven one, plenty of computational requirements have been eliminated. The event-driven simulation method is called “lazy evaluation method” because it delays the evaluation computation as much as possible. The lazy evaluation simulation method requires access to the synaptic matrix stored in main memory, both row-wise and column-wise. A presynaptic spike triggers an update of a single row in the synaptic matrix, while a post-synaptic spike triggers an update of a single column. Today's memory architecture, such as DRAM, cannot handle two orthogonal directional access patterns of the same block of continuous data without sacrificing efficiency. Such access patterns will also affect the efficiency of the cache system. Therefore, we propose to remove the column update procedure and to merge the column and row update. In this way, we avoid entirely the dual memory access pattern that degrades the efficiency of the BCPNN simulation. Furthermore, by carefully designing our strategy, we can also reduce the demand in storage requirement and memory access, while increasing the overall performance. We remind readers that even though the BCPNN is the optimization target, our method is not restricted to this learning rule. Any Hebbian based neural network learning rule could potentially be optimized with a slightly modified version of our strategy.

The rest of the paper is organized as follows: section 2 explains the original BCPNN learning rule, points out the memory access problem, proposes the alternative method that resolves the problem, and performs an error analysis for the proposed method. Section 3 demonstrates the benefits of the proposed method in terms of both memory storage requirements and performance. Finally, section 4 summarizes the paper and addresses the potential of the proposed method.

## 2. Methodology

In this section, we introduce the lazy evaluation BCPNN simulation strategy and highlight the memory access problem. To overcome this problem, we propose an optimization technique that tackles the memory access problem. We also present a detailed analytical and experimental error analysis that shows the probability of introducing intolerable errors is negligible. Since we will not drastically modify the BCPNN learning rule in this paper, we will just present its essence. Readers can find the complete and detailed description in Vogginger et al. (2015).

### 2.1. The BCPNN Learning Rule

BCPNN is a type of artificial neural network whose learning rule is derived from Bayes' theorem. It strengthens or weakens the connectivity/weight between pre- and post-synaptic neurons based on their co-activation. A correlated pre- and post-synaptic activity gives a positive weight, whereas an anti-correlation gives a negative weight. The connectivity/weight is calculated by Bayes' theorem based on the measured firing probability of pre- and post-synaptic neurons.

To mimic the biological columnar cortical structure (Buxhoeveden and Casanova, 2002), BCPNN considers minicolumn units (MCUs) as its basic units representing the aggregation of about a hundred neurons. Many MCUs form a hypercolumn unit (HCU) representing the biological hypercolumn structure (Hubel and Wiesel, 1974). MCUs in each HCU compete with each other in a soft winner-take-all (soft WTA) fashion, representing the net effects of excitatory and inhibitory connections among neural cells, as shown in Coultrip et al. (1992) and Lundqvist et al. (2006). We use the notation *H* × *M* to represent a BCPNN configuration that consists of *H* HCUs, and each HCU includes *M* MCUs. Usually, we have the constraint *H* ≥ *M*. *H* can be increased arbitrarily without an upper limit. *M*, on the other hand, has a limit of *M* = 100. Therefore, the network is growing purely due to the growth of the amount of HCUs for big networks. For example, a typical human cortex comparable BCPNN configuration is ~2 · 10^6^ × 100 (Johansson and Lansner, 2007).

**Figure 1:**
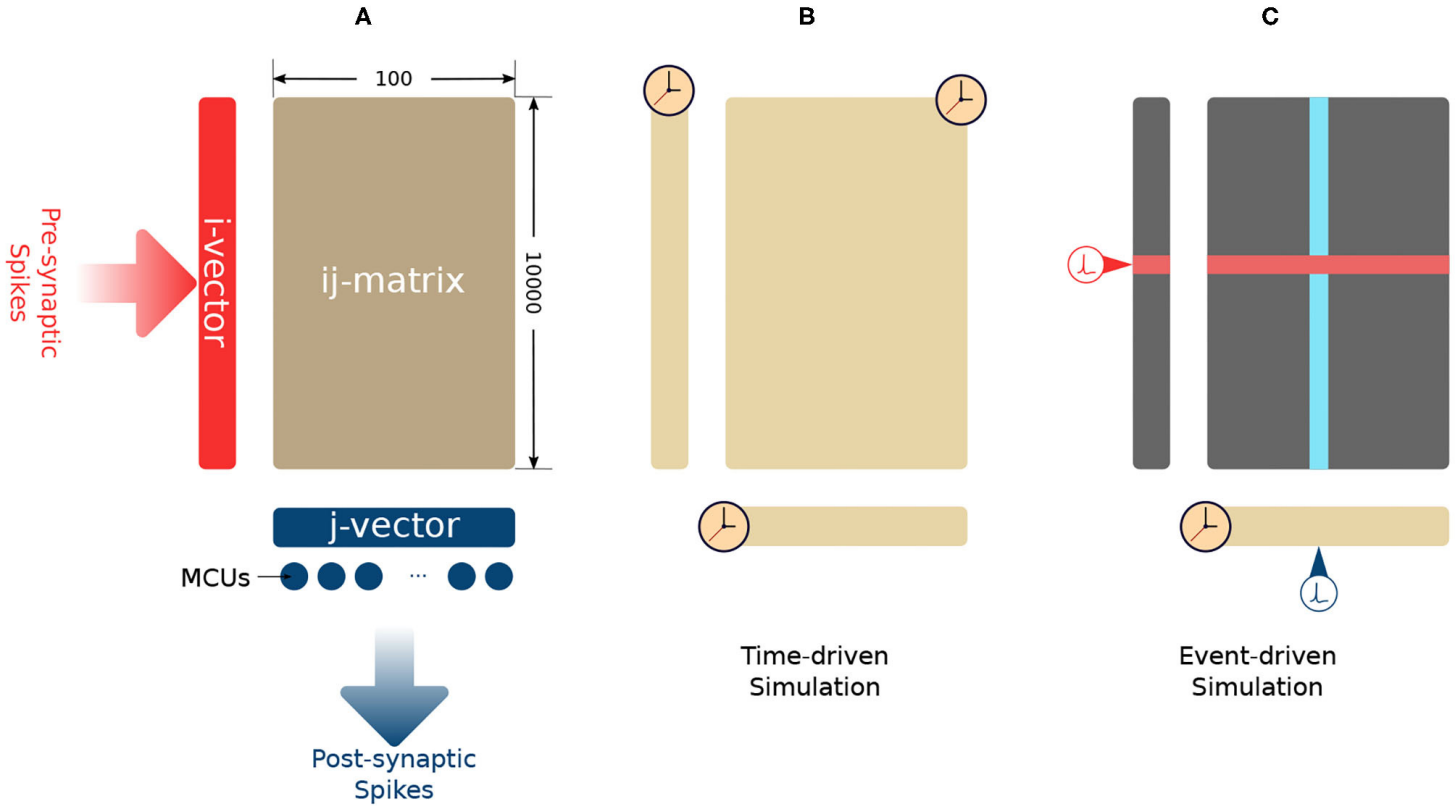
**(A)** Synaptic connections in an HCU. **(B)** Time-driven simulation method. The update of every trace is triggered by simulation time. **(C)** Lazy evaluation method. The update of *j*-vector (yellow) is triggered by time. The row update (red) is triggered by presynaptic spikes. The column update (blue) is triggered by post-synaptic spikes.

Each MCU could connect to an HCU via a sparse patchy connection (Meli and Lansner, 2013). Synapses are formed due to these connections. Fully connected big networks are very costly in terms of storage and computation. A parameter *C* constraints the amount of possible incoming connection slots of each HCU. The parameters *C* and *M* define the shape of its synaptic matrix. In each human cortex comparable HCU, a 10^4^ × 100 synaptic matrix is used to store the intermediate synaptic traces as well as the synaptic weights, as shown in Figure 1A. This HCU configuration uses *C* = 10^4^ incoming connections and *M* = 100 MCUs. On the presynaptic side (left side) of the synaptic matrix, an *i*-vector of size *C* = 10^4^ is used to store presynaptic traces *z*_*i*_, *e*_*i*_, and *p*_*i*_. On the post-synaptic side (bottom side), a *j*-vector of size *M* = 100 is used to store post-synaptic traces *z*_*j*_, *e*_*j*_, and *p*_*j*_. The synaptic matrix is also called the *ij*-matrix because it stores the synaptic traces *e*_*ij*_, *p*_*ij*_, and *w*_*ij*_.

Following Bayes' theorem, the BCPNN learning rule requires the probability estimation of both pre- and post-synaptic events (spikes). Such probability estimation is obtained via a chain of low-pass filters applied on the pre- and post-synaptic spikes (*s*_*i*_ and *s*_*j*_). For example, the presynaptic filter chain is *s*_*i*_ → *z*_*i*_ → *e*_*i*_ → *p*_*i*_. Similar filter chains are also used to generate *p*_*j*_ (*s*_*j*_ → *z*_*j*_ → *e*_*j*_ → *p*_*j*_) and *p*_*ij*_ (*z*_*i*_ * *z*_*j*_ → *e*_*ij*_ → *p*_*ij*_). The last three traces in the chain (*p*_*i*_, *p*_*j*_, and *p*_*ij*_) represent the firing probability for presynaptic spikes, post-synaptic spikes as well as pre- and post-synaptic spike co-activation. The synaptic weight is then computed by combining these three traces. These filters are implemented by ordinary differential equations (ODEs). Equation (1) is an example of such ODEs where *X* is the input, *Y* is the output, and τ is the time constant. These ODEs can be easily computed by Euler's method (Griffiths and Higham, 2010) in time-driven simulation.

(1) $$\begin{array}{l} \tau \frac{dY}{dt}=X-Y \end{array}$$

As a numeric method, Euler's method requires updating all the traces whenever the simulation time is forwarded one simulation step Δ*t*, as shown in Figure 1B. The synaptic plasticity is caused by the interaction between pre- and post-synaptic spikes. These spikes drive the change of synaptic weights. The update of synaptic traces is not necessary when the spikes are absent. As long as we account for the decay of all the traces when a pre- or post-synaptic spike comes, then the network's mathematical behavior will be the same as for time-driven simulation. As shown in Figure 1C, a lazy evaluation method only updates part of *i*-vector and *ij*-matrix when there is a triggering spike. The current value of traces will be calculated analytically based on the time difference between the current simulation step and the time when the previous spike came. In this paper, we use the “Analytical I method” in Vogginger et al. (2015) as the baseline and refer it as the “lazy evaluation method.” Equation (2) shows the complete set of equations of the lazy evaluation method. These equations are used for calculating of *p*_*i*_ and *p*_*ij*_. The calculation of *p*_*j*_ remains as time-driven. The detail of the lazy evaluation method is not in the scope of this paper, readers can find the proof of equivalence in Vogginger et al. (2015).

(2) $$\begin{array}{l} \begin{array}{c} z_{i}\left(t\right)=z_{i}\left(t^{last}\right)\cdot e^{-\frac{\Delta t}{\tau_{z_{i}}}}+s_{i}\left(t\right)\\ e_{i}\left(t\right)=e_{i}\left(t^{last}\right)\cdot e^{-\frac{\Delta t}{\tau_{e}}}+\;a_{i}\;\left(e^{-\frac{\Delta t}{\tau_{z_{i}}}}-e^{-\frac{\Delta t}{\tau_{e}}}\right)\;z_{i}\left(t^{last}\right)\\ p_{i}\left(t\right)=p_{i}\left(t^{last}\right)\cdot e^{-\frac{\Delta t}{\tau_{p}^{*}}}+a_{i}b_{i}\;\left(e^{-\frac{\Delta t}{\tau_{z_{i}}}}-e^{-\frac{\Delta t}{\tau_{p}^{*}}}\right)\;z_{i}\left(t^{last}\right)\\ \;+\left(e_{i}\left(t^{last}\right)-a_{i}z_{i}\left(t^{last}\right)\right)\;c\;\left(e^{-\frac{\Delta t}{\tau_{e}}}-e^{-\frac{\Delta t}{\tau_{p}^{*}}}\right)\\ e_{ij}\left(t\right)=e_{ij}\left(t^{last}\right)\cdot e^{-\frac{\Delta t}{\tau_{e}}}+{\;a}_{ij}\;(e^{-\frac{\Delta t}{\tau_{z_{ij}}}}-e^{-\frac{\Delta t}{\tau_{e}}}){\;z}_{i}\left(t^{last}\right)z_{j}\left(t^{last}\right)\\ p_{ij}\left(t\right)=p_{ij}\left(t^{last}\right)\cdot e^{-\frac{\Delta t}{\tau_{p}^{*}}}+a_{ij}b_{ij}\;(e^{-\frac{\Delta t}{\tau_{z_{ij}}}}-e^{-\frac{\Delta t}{\tau_{p}^{*}}}){\;z}_{i}\left(t^{last}\right)z_{j}\left(t^{last}\right)\\ \;+\left(e_{ij}\left(t^{last}\right)-a_{ij}z_{i}\left(t^{last}\right)z_{j}\left(t^{last}\right)\right)\;c\;(e^{-\frac{\Delta t}{\tau_{e}}}-e^{-\frac{\Delta t}{\tau_{p}^{*}}}) \end{array} \end{array}$$

where,

$$\begin{array}{l} a_{i}=\frac{\tau_{z_{i}}}{\tau_{z_{i}}-\tau_{e}}\;b_{i}=\frac{\tau_{z_{i}}}{\tau_{z_{i}}-\tau_{p}^{*}}\;c=\frac{\tau_{e}}{\tau_{e}-\tau_{p}^{*}}\\ \tau_{z_{ij}}={\left(\frac{1}{\tau_{z_{i}}}+\frac{1}{\tau_{z_{j}}}\right)}^{-1}\;a_{ij}=\frac{\tau_{z_{ij}}}{\tau_{z_{ij}}-\tau_{e}}\;b_{ij}=\frac{\tau_{z_{ij}}}{\tau_{z_{ij}}-\tau_{p}^{*}} \end{array}$$

Each MCU works as a leaky integrator that integrates the input spike effects (*s*_*i*_·*w*_*ij*_) in terms of membrane potential. These MCUs in the same HCU then compete with each other based on their membrane potential in soft-WTA fashion. The soft-WTA process normalizes their membrane potential and generates a relative firing rate *o*_*j*_. If the soft-WTA process has selected a winning MCU, the *o*_*j*_ of the winning MCU will be approaching 1. The rest losing MCUs will be suppressed to an *o*_*j*_ approaching 0. That means the winning MCU will have a higher probability of firing than the rest. If there is no clear winner, all the MCUs will have nearly uniform *o*_*j*_ after the soft-WTA process. In this case, the MCUs will have a relatively low but equal firing probability. The *o*_*j*_ is then scaled to match the firing rate range in order to give the final firing rate *r*_*j*_, Equation (3). The firing rate range is between 0 and the maximum firing rate (*r*_*max*_), where the maximum firing rate is usually set to 0.1.

(3) $$\begin{array}{l} r_{j}=r_{max}\cdot o_{j} \end{array}$$

Finally, to generate a spike from the firing rate *r*_*j*_, a Poisson spike generator (Dayan and Abbott, 2001) shown in Equation (4) is employed. A uniformly distributed random number *x* is generated every time and compared to *r*_*j*_. If *r*_*j*_ is bigger than *x*, the MCU fires.

(4) $$\begin{array}{l} s_{j}=\{\begin{array}{cc} 1, & \text{if }r_{j}>x,x\sim U\left(0,1\right)\\ 0, & \text{otherwise} \end{array} \end{array}$$

### 2.2. Memory Access Problems of Lazy Evaluation Method

By adopting the lazy evaluation method, the massive memory access and computation demand from the time-driven simulation can now be avoided. However, the lazy evaluation method is not perfect. Its most significant issue is the dual-memory access pattern. Both pre- and post-synaptic spikes trigger the update events of synaptic traces. Depending on the type of spikes, either a row or a column of the synaptic matrix is fetched. The row or column is sent to the computation unit to be updated and stored back. The lazy evaluation implies that memory storage should efficiently support both row-wise and column-wise access mode to achieve high throughput. However, such a requirement is not feasible for modern DRAM and cache hierarchy of supercomputers.

Modern DRAM and cache respond to every *READ* and *WRITE* operation with a whole row of their data to increase efficiency. Isolated single cell access pattern is not friendly for DRAM and cache. To access a single data cell, they need to fetch or store a whole row of data. Since other parts of the row are useless, to operate on them is a waste of time and energy.

The lazy evaluation method with both row-wise and column-wise access patterns can be a great challenge for the DRAM and cache system. In Stathis et al. (2020), the author tries to customize the DRAM architecture to make it more adaptable to the BCPNN access pattern. However, due to the nature of DRAM technology, even by heavy customization, it still sacrifices the DRAM performance to support lazy evaluation. In this work, we propose a modified lazy evaluation method that eliminates the column-wise memory access pattern. This optimization makes the BCPNN learning rule DRAM and cache access friendly. We refer to the modified algorithm as *Column Update Elimination*, or *CUE* for short.

The CUE method not only solves the dual memory access problem but also eliminates the demand for memory access by the column update process. Even with the lazy evaluation method, which has already dramatically reduced the memory access, the memory access demand is still huge for a large BCPNN. To put it into context, a human cortex comparable BCPNN has a 2 · 10^6^ × 100 configuration. Such a network needs to access, on average, 10,000 rows and 100 columns of synaptic storage in every second per HCU. In total, it will require 200 TB of data traffic for the whole network per second. The amount of data required by 100 rows and 1 column is the same, around 240 KB/HCU, because the shape of the synaptic matrix is 10,000 × 100 for the human cortex comparable BCPNN. By applying CUE, half of the memory access demand due to column update is eliminated.

### 2.3. Column Update Elimination (CUE)

In this section, we modify the original BCPNN lazy evaluation method by eliminating the more expensive column update. In each subsection, we will introduce one important modification and explain in detail its mechanism. The main idea of this modification is to avoid column-wise access to DRAM and cache by removing the column update. The row update triggered by the presynaptic spike updates the synaptic weight, which directly influences the spike generation. On the other hand, the column update only affects the state of the synaptic traces and can be delayed. The column update can be removed, and its calculations can be integrated with the row update.

#### 2.3.1. Ideal CUE

**Figure 2:**
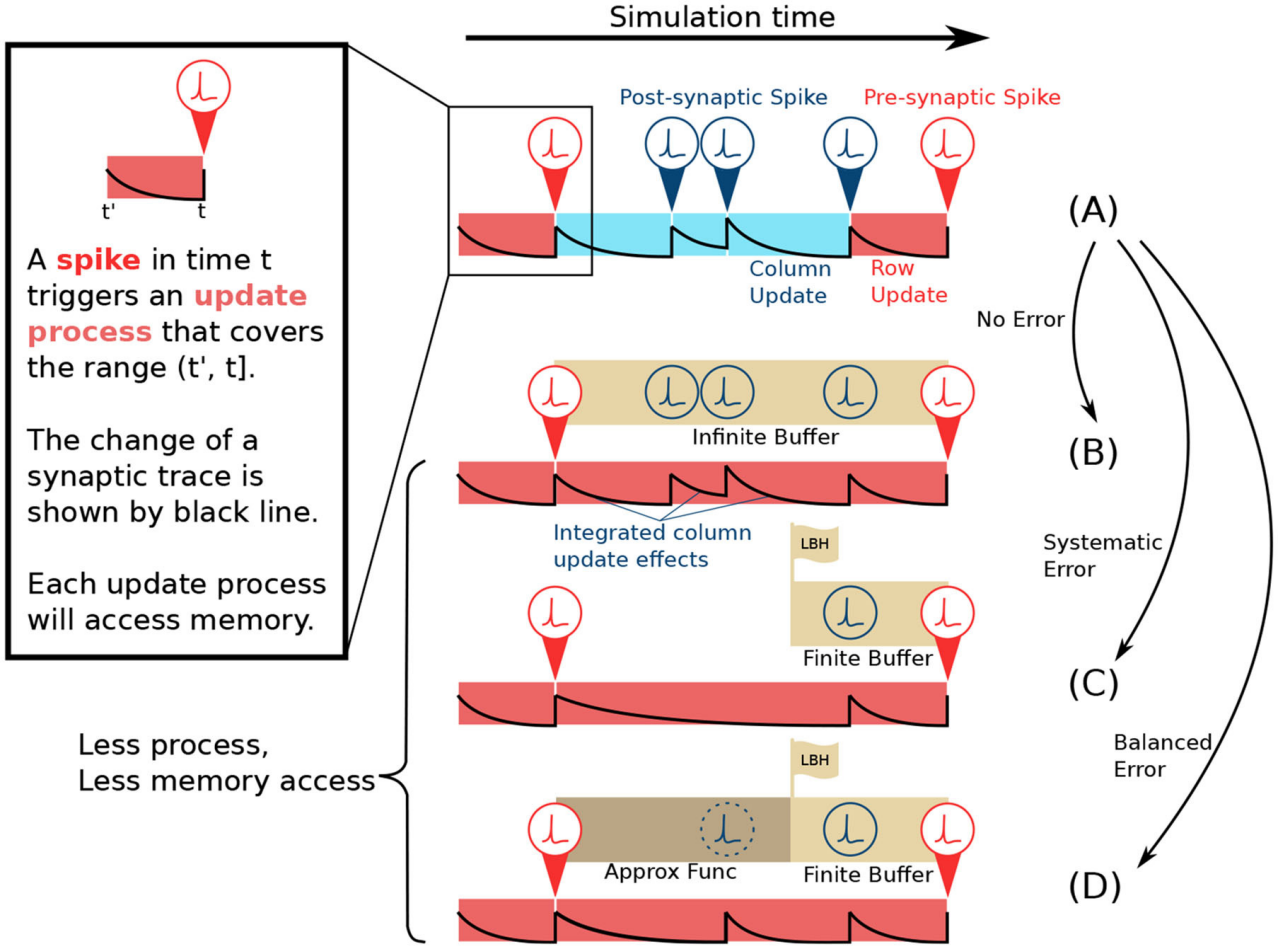
Comparison of lazy evaluation method and CUE simulation strategy. **(A)** Lazy evaluation method. The update is performed either by presynaptic spike triggered row update (red) or post-synaptic spike triggered column update (blue) process. **(B)** CUE method with infinite buffer. The update is performed only by the presynaptic spike triggered row update. The row update is modified to incorporate the effects of the original column update. The change of synaptic trace also considers the post-synaptic spikes. **(C)** CUE method with finite buffer. Finite buffer might discard spikes and cause a systematic error. The change of synaptic trace has been altered due to the discarded spikes. **(D)** CUE method with finite buffer and approximation function. The approximation function will cover the range that can't be covered by the spike buffer. It will introduce a balanced error which is preferable.

As shown in Figure 2A, in the lazy evaluation method, each cell in the *ij*-matrix will be updated either by row update (red) or column update (blue). A spike triggers each update event. The update procedure includes three tasks: Load data from memory, perform the computation, and store data back to memory. Row update is triggered by presynaptic spikes *s*_*i*_ (red), while column update is triggered by post-synaptic spikes *s*_*j*_ (blue). The example in Figure 2A, shows the update event of a single cell in the *ij*-matrix. From left to right, its traces are updated by a row update, followed by 3 column updates, and another row update. Each update only covers the range from its trigger point back until the last spike event.

If an infinite buffer that records all the post-synaptic spikes is available, the column update can then be eliminated, as shown in Figure 2B. Each cell in the *ij*-matrix will only be updated by a row update (red). The row update will cover the range from its trigger point back until the last presynaptic spike event. However, the row update process in Figure 2B is different from Figure 2A. The new row update process emulates the computation of the original column updates thanks to the buffer that keeps the record of all post-synaptic spikes. Compared to the original lazy evaluation method, the CUE row update reduces the amount of memory access leaving only the row-wise memory access patterns.

The CUE method with an infinite sized buffer will not introduce any additional error, as all the computation required by the lazy evaluation method is still performed. It only changes the point in time when each computation happens. The CUE does not reduce the amount of computation. It only reduces the amount of memory access. The integration of column update effects is summarized by Algorithms 1 and 2.

**Algorithm 1:**
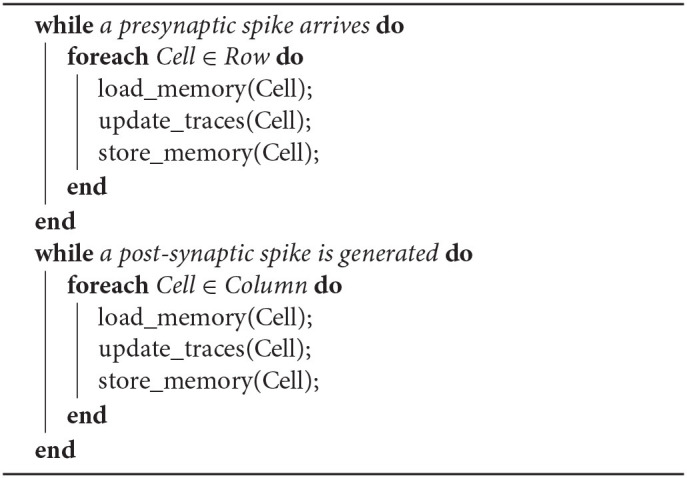
Lazy Evaluation Method

**Algorithm 2:**
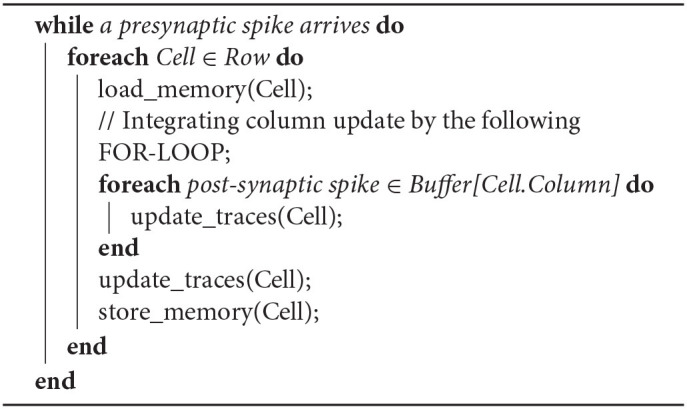
Ideal CUE Method

#### 2.3.2. CUE With Finite Sized Buffer

To have an infinite buffer that records every post-synaptic spike is impractical. In a more realistic case, the infinite buffer can be substituted by one with limited size, as shown in Figure 2C. The edge of the buffer is called the look-back horizon (*LBH*). A spike history buffer with size *L* can only respond to the request for *s*_*j*_(*t*) when *t* is later in time than the LBH. The information from spike events that are earlier in time than LBH is lost.

As shown in the example in Figure 2C, two post-synaptic spikes that are beyond the LBH are discarded by the spike history buffer, due to its limited size. Therefore, the row update computation, that emulates the effects of the intermediate column updates is different from the original lazy evaluation computation. Such behavior will introduce systemic errors since spikes are solely dropped. The post-synaptic spike train observed by the row update in Figure 2C will always have fewer spikes than the spike train in Figure 2A. This systematic error is undesirable because it will accumulate strictly positively or negatively. We need a mechanism that could introduce balanced errors so that they can potentially cancel each other.

#### 2.3.3. Approximation Function

To avoid a systematic error, we propose an approximation function to predict the spikes beyond the LBH, as shown in Figure 2D. Since the spike history is lost, due to the limited buffer size, the prediction is the only option when the information of *s*_*j*_ beyond LBH is needed. An approximation function is defined as $H:{\mathbb{N}}^{0}\times 𝔻\to 𝔹;\left(t,m\right)\mapsto s$. where *t* ∈ ℕ^0^ is the simulation step that needs prediction, *m* ∈ 𝔻 is whatever extra information required by the approximation function, and *s* ∈ 𝔹 is the boolean variable indicating whether a spike should be generated or not.

Errors will be introduced if the approximation function is not an oracle that always gives a correct prediction. In the next section, we will discuss various approximation functions in detail and analyze their error bounds. A good approximation function should be computationally light and be able to reduce the error, compared to the scenario when all the spikes beyond LBH are dropped. Any errors introduced by the approximation function should be introduced in a balanced way to avoid error accumulation.

#### 2.3.4. Alternative Approach in Literature

Knight et al. (2016) has reported a similar method to remove the BCPNN column update by introducing a finite buffer. The work does not mention how the expired spikes are handled. If the expired spikes are just dropped, the BCPNN will suffer from systematic error.

Another paper by the same author (Knight and Furber, 2016) has reported a “flushing event” mechanism to avoid the loss of spikes when using a finite-sized buffer for STDP. The flushing event method used in STDP is not very friendly for BCPNN. A presynaptic neuron triggers a flushing event when it hasn't been active for a fixed time (usually spike buffer size *L*) and triggers an update of a row in the synaptic matrix. In BCPNN, when the internal representation is stable, the active rows (very small proportion of the synaptic matrix) are also stable. For example, in a 100 × 100 network with 10,000 rows in total, the number of active rows is statistically always the same 100 rows due to the bursty property of spike trains. Therefore, presynaptic neuron triggered flushing events will force almost a complete full matrix update every *L* ms. In this paper, we have optimized the flushing event method to be triggered by post-synaptic spikes. It forces a column update whenever a post-synaptic spike is shifted out from the spike buffer to guarantee that no spike is lost. The post-synaptic triggered version statistically only updates a synaptic column instead of the whole synaptic matrix every *L* ms. Different from our approach which uses approximation function to predict the spikes, the flushing event method is exact and will not introduce error. But the price would be keeping the inefficient column update process.

In section 3, we implement the flushing event method on BCPNN and compare both the flushing event method and our CUE method against the baseline lazy evaluation method. Readers will see that by removing the column update process, CUE method outperforms the flushing event method in terms of both storage and performance.

### 2.4. Error Analysis

In this section, we analyze the error introduced by the CUE method. The error discussed in this paper refers to the relative error of synaptic weight *w*_*ij*_ caused by wrong post-synaptic spike predictions. We choose *w*_*ij*_ because it is the final synaptic variable that influences the spike generation. The error is treated only at the evaluation points since the row update only happens at these timing points. The error is defined by Equation (5). For simplicity, we denote the evaluation of *err*(*t*) at the evaluation points simply by *err* in later text. In this section, we bound the probability of intolerable error. Two small threshold numbers, ϵ and δ, are defined for such error bound. Equation (6) describes the *err*, as a function of ϵ and δ.

(5) $$\begin{array}{l} \begin{array}{c} err(t)=\{\begin{array}{cc} \left|\frac{w_{ij}(t)-w_{ij}^{pred}(t)}{w_{ij}(t)}\right|, & \text{if }s_{i}(t)=1\\ 0, & \text{otherwise} \end{array} \end{array} \end{array}$$

(6) $$\begin{array}{l} \begin{array}{c} P\left(err>\epsilon |L,H\right)=P\left(t-t^{\prime }>L\right)\cdot P\left(err>\epsilon |H\right)\leq \delta \end{array} \end{array}$$

Equation (6) represents the probability of having an error that exceeds the threshold ϵ, under the scenario that a spike buffer of size *L* is used together with an approximation function H. This probability is bounded by δ. If we prove that δ is sufficiently small, we can assert that the BCPNN behavior will not diverge from the original simulation strategy. We do not intend to mathematically bound the error to a definite number that works for every corner case because such an approach is very pessimistic and does not reflect typical operational scenarios.

The probability P(err>ϵ|L,H) can be expanded as two terms. The two terms describe the conditions: (1) The last presynaptic spike fires beyond the look-back horizon (LBH), and (2) the approximation function prediction gives an intolerable error. In Equation (6), *t* is the current evaluation time when a presynaptic spike is observed, and *t*′ is the time when the last presynaptic spike occurs for the same synaptic cell.

In the following sections, we first discuss the spike firing rate distribution to form the foundation for the probability-bound calculation. Then we calculate the probability of a presynaptic spike firing beyond the LBH, *P*(*t* − *t*′ > *L*). After that, we present two approximate functions—the static and adaptive approximation function. We establish the probability of introducing errors via the two approximation functions, P(err>ϵ|H). Finally, we summarize all the strategies and compute the overall error probability bound.

#### 2.4.1. Spike Firing Rate Distribution

The average spiking frequency in higher-order (memory/cognitive) cortical areas is very low, likely around 0.1 Hz (Lennie, 2003). When experimenters record active neurons, they typically have spiking frequency up to 100 Hz with an average of around 20 Hz. An MCU in BCPNN does not directly represent every single neuron. Instead, it mimics the behavior of a minicolumn of some hundred neurons, of which, only a handful (5–10) big layer 5 pyramidal cells communicate outside the HCU. So we estimate that the maximum instantaneous firing frequency of an MCU is 5 × 20 = 100 Hz. Therefore, the maximum firing rate at each simulation step *r*_*max*_ is set to 0.1 when the simulation step Δ*t* is set to 1 ms.

An *H* × *M* BCPNN has an activity level α ∈ [0, 1]. It indicates the number of active HCUs in this BCPNN is α*H*. An active HCU is an HCU with a clear winning MCU and some losing MCUs after the soft Winner-Take-All (soft WTA) process while an inactive HCU has only MCUs that are neither winners nor losers, see section 2.1. Winning MCUs will have a high firing rate (close to *r*_*max*_), losing MCUs have a low firing rate (close to 0), and MCUs in the inactive HCU will have uniform firing rate (close to $\frac{r_{max}}{M}$). Usually, a BCPNN should not have the majority of its HCUs inactive as those HCUs would not be able to learn.

**Figure 3:**
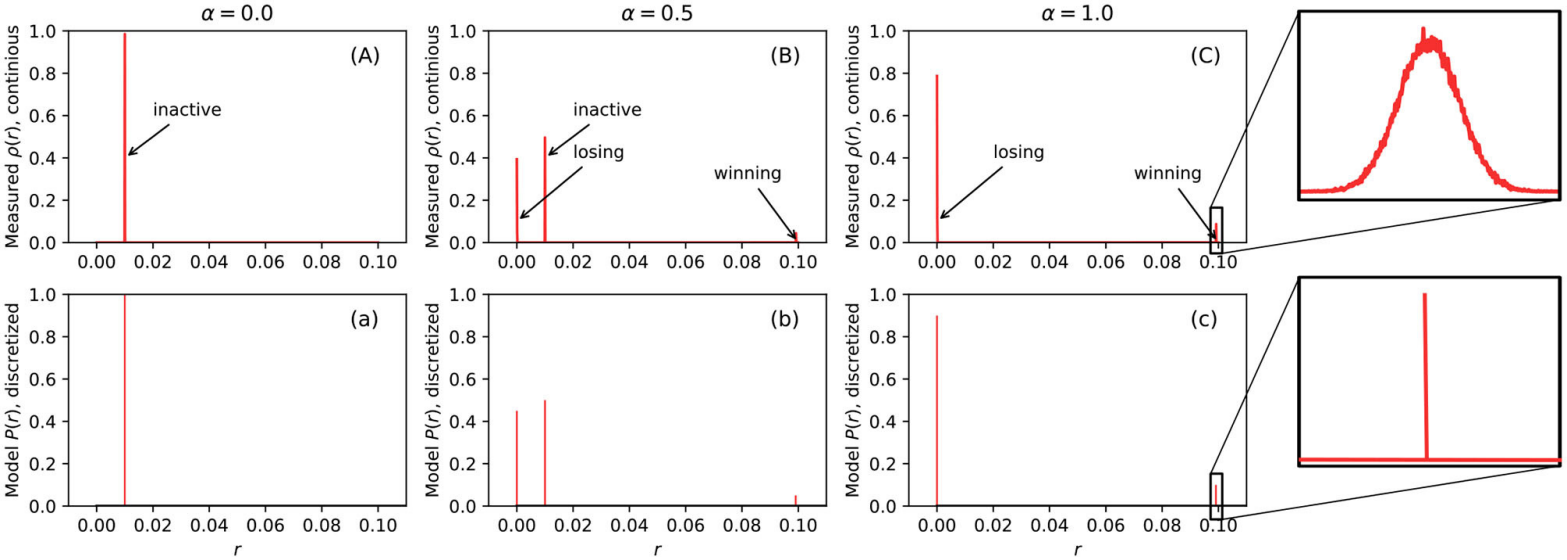
The measured probability distribution function of firing rate ρ(*r*) (first row, **A–C**) and the model of probability distribution of firing rate *P*(*r*) (second row, **a–c**). The first row is continuous function while the second row is discretized function. The horizontal axis of all sub figures ranges from 0 to 0.1 because *r*_*max*_ = 0.1 and both ρ(*r*) and *P*(*r*) are 0 when *r* > *r*_*max*_.

The first row of Figures 3A–C, show examples of measured firing rate probability distribution function ρ(*r*) of a 10 × 10 BCPNN. From the figure, we can see that the distribution of firing rates is very dense in some extremely narrow areas. If we zoom in, as shown in sub-figure (C), we can observe a narrow bell-curve shaped distribution. The cause of each peak is marked by text beside all the sub-figures in the first row (A), (B), and (C). For example, sub-figure (A) has only one peak caused by the MCUs in the inactive HCUs, since when α = 0, all HCUs are inactive. In sub-figure (C), two peaks are caused by losing and winning MCUs inside the active HCUs, since when α = 1, all HCUs are active, hence there is a winning MCU and some losing MCUs. At last, when α = 0.5, it is the combination of the previous two scenarios. The height of each pulse is determined by the amount of MCUs that cause it. If we compare the peak height caused by losing and winning MCUs, we can see that the losing peak is much higher than the winning one, because there are nine times more losing MCUs than the winning MCUs in active HCUs due to the soft-WTA process.

(7) $$\begin{array}{l} \begin{array}{c} P\left(r\right)=\{\begin{array}{cc} \alpha \frac{M-1}{M}, & \text{if }r=r_{l},\\ \alpha \frac{1}{M}, & \text{if }r=r_{w},\\ 1-\alpha , & \text{if }r=r_{s},\\ 0, & \text{otherwise} \end{array} \end{array} \end{array}$$

We can use impulse functions δ(·) to model the probability distribution function (PDF) of firing rate ρ(*r*). By using a δ function, the probability distribution of the firing rate is discretized. Therefore, we can directly use *P*(*r*) shown in Equation (7) to describe the discretized events. *P*(*r*) is shown in the second row of Figures 3a–c. The position of these peaks in the model is determined by the average firing rate *r*_*l*_ of losing MCUs in active HCU, the average firing rate *r*_*w*_ of winning MCUs in active HCU, and the average firing rate *r*_*s*_ of MCUs in inactive HCU. The constants *r*_*l*_, *r*_*w*_, and *r*_*s*_ are measured directly from BCPNN simulations. Since the firing rate is determined by the soft-WTA process inside a single HCU, the only factor that influences these constants is the number of MCUs in each HCU (*M*), normally ranging from 10 to 100. Different BCPNN applications might have a different value for these constants. An investigation of both feed-forward and recurrent BCPNN suggests that *r*_*l*_ and *r*_*w*_ are very concentrated and close to 0 and *r*_*max*_, respectively. The value of *r*_*s*_ is analytically determined by assuming all MCUs in inactive HCU have a uniform firing rate. To represent the general firing rate distribution, we use a set of constants that we obtained from the simulation of the BCPNN as an associative memory via a BCPNN GPU simulator (Herenvarno, 2019). These constants are listed in Table 1.

**Table 1:** Firing rate constants.

**M**	**10**	**20**	**30**	**40**	**50**
*r*_*l*_	0.0000990	0.0000981	0.0000972	0.0000963	0.0000954
*r*_*w*_	0.0991090	0.0981361	0.0971812	0.0962443	0.0953254
*r*_*s*_	0.0100000	0.0050000	0.0033333	0.0025000	0.0020000
**M**	**60**	**70**	**80**	**90**	**100**
*r*_*l*_	0.0000945	0.0000936	0.0000927	0.0000918	0.0000909
*r*_*w*_	0.0944245	0.0935416	0.0926767	0.0918298	0.0910009
*r*_*s*_	0.0016667	0.0014286	0.0012500	0.0011111	0.0010000

We can now calculate the expectancy of *r* based on Equation (7) and the constants in Table 1. The Equation (8) shows the method to calculate such expectancy. It shows that the expectancy is always very close to *r*_*s*_ regardless of α and *M*.

(8) $$\begin{array}{l} \begin{array}{c} \langle r\rangle =\underset{r\in \left\{r_{l},r_{w},r_{s}\right\}}{\sum }r\cdot P\left(r\right)\\ \;=\alpha \left(r_{w}\frac{1}{M}+r_{l}\frac{M-1}{M}\right)+\left(1-\alpha \right)r_{s}\\ \;\approx \alpha \left(r_{max}\cdot \frac{1}{M}+0\cdot \frac{M-1}{M}\right)+\left(1-\alpha \right)\frac{r_{max}}{M}\\ \;=\frac{r_{max}}{M}=r_{s} \end{array} \end{array}$$

From the recordings of cortical memory systems, it is clear that spikes typically come in the form of bursts (Lundqvist et al., 2016). The BCPNN implementation is optimized for this kind of behavior as its internal representation does not change too frequently with respect to the simulation step. Therefore, when analyzing spike sequences, we assume that the firing probability does not change for the whole spike sequence in the observing period (typically <200 ms). When at some simulation step, we observe a firing rate *r*, it is almost certain that at one step earlier, the firing rate was also *r*.

#### 2.4.2. Spike History Buffer

**Figure 4:**
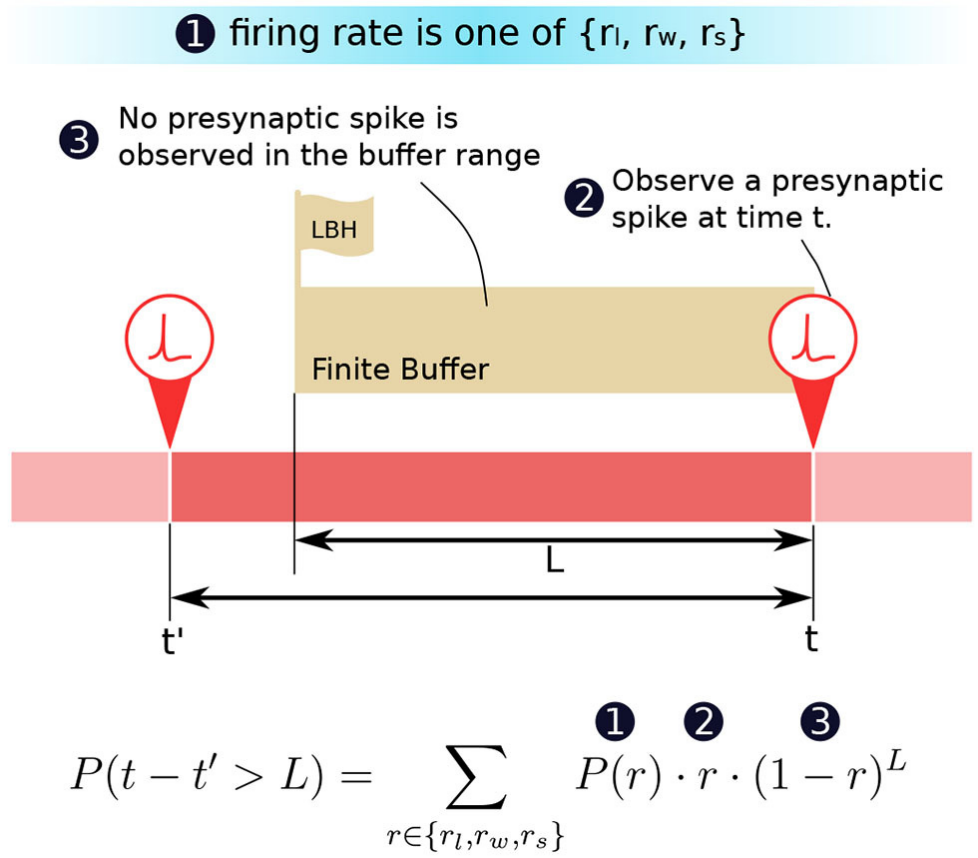
The decomposition of the probability *P*(*t* − *t*′ > *L*).

We are now in a position to formulate how the probability *P*(*t* − *t*′ > *L*) can be computed in terms of the firing rate *r* and its probability distribution *P*(*r*). We know that (1) in a period, the probability of the presynaptic neuron firing at a particular firing rate *r* is *P*(*r*). (2) The probability of a presynaptic spike appearing at instance *t* is then equal to the firing rate *r* of that period. When there is indeed a presynaptic spike, a row update will trigger the proposed CUE scheme. (3) Given point (2) we can deduce that the probability that no spikes appeared for the previous *L* ms is (1 − *r*)^*L*^. If *t*′ is the instance when the row was last updated, then the probability of having (*t* − *t*′) > *L* is exactly the combination of the three conditions described above. Figure 4 illustrates the condition and time relation.

**Figure 5:**
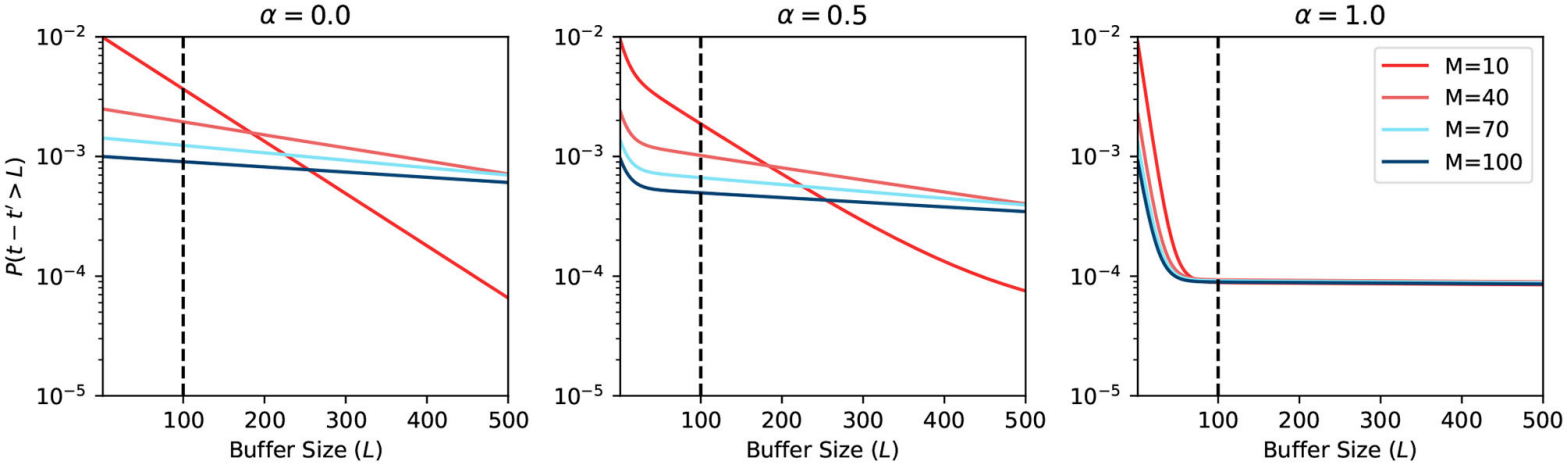
Probability of looking beyond LBH.

Figure 5 shows the plot of such a probability based on the equation in Figure 4. The horizontal axis represents the buffer size *L*. The vertical axis is in logarithmic scale and represents the probability of requiring to look beyond the LBH. When α = 0, all MCUs will have a uniformly distributed firing rate *r*_*s*_. Thus, all the curves in the first sub-figure are straight lines. When α = 1, there are two types of MCUs that fire under *r*_*l*_ or *r*_*w*_, which gives the curve two distinct parts. The first part of the curve is dominated by the effects of winning MCUs, where the probability drops very fast. The second part is almost flat, which is dominated by losing MCUs. The sub-figure in the center with α = 0.5 is the combination of the above scenarios. The BCPNN configuration parameter *M* also affects the shape of the curve. When *M* is bigger, the *r*_*s*_ will become closer to 0, making the probability smaller. The overall average firing rate of active HCUs is also roughly equaled to *r*_*s*_. That is why all three sub-figures, though with different α, have curves starting at the same points, which are determined by *r*_*s*_.

The most common BCPNN applications have most of their HCUs being active. Thus, the majority of their MCUs are either winning or losing after the soft-WTA process. According to the third sub-figure in Figure 5, we choose the *L* = 100 as the size of the spike buffer since it already passes the turning point. Further increasing the buffer size will not dramatically decrease the error probability.

#### 2.4.3. Approximation Function

We have briefly discussed the approximation function and have explained why imperfect approximation function will introduce errors. Although it is impossible to avoid introducing errors with any imperfect approximation function, a well-designed approximation function can introduce small errors in a balanced manner. Therefore, we need to choose a proper tolerance ϵ so that the error bound can reflect the approximation function's predictive ability. In this work, we choose ϵ = 1% as the threshold. It is a practical way to consider the error to be much less than the actual value in insensitive systems, such as neural networks. It thus could guarantee that the behavior of the BCPNN remains unaffected. We use ϵ to calculate the error bound of approximation functions.

It is very complex to analytically find out the error propagation from the firing rate *r* to synaptic weights *w*_*ij*_, as discussed in section 2.1. The calculation of *w*_*ij*_ includes many low-pass filters, random number generation, and complex arithmetic operations, such as logarithm. Instead, we opt to find out P(err>ϵ|H) by experimental simulation for each type of approximation functions that have been developed. Each experiment has been repeated for more than 10^5^ times to guarantee the generality of the collected statistics.

##### 2.4.3.1. Static approximation function

In this subsection, we propose a type of approximation function that we call static. The static approximation function $H_{s}$ is defined in Equation (9), where x~U(0,1) is a uniformly distributed random number generated at each prediction. This function is static because it always uses a constant firing rate to make a prediction. We use the *s* subscript to indicate that the function is static.

(9) $$\begin{array}{l} H_{s}:{\mathbb{N}}^{0}\to 𝔹,\text{so that }H_{s}:t\mapsto \left(x<r_{s}\right) \end{array}$$

To maximize the probability for long term correct prediction, the predicted firing rate has to match the true firing rate. However, a spike can be generated according to any of *r*_*l*_, *r*_*w*_, and *r*_*s*_. Therefore, the true firing rate is not constant. We choose *r*_*s*_ to be the static predicted firing rate is because the expectancy of *r* is 〈*r*〉 = *r*_*s*_. It will statistically introduce both positive and negative errors since it does not exactly match the true firing rate.

**Figure 6:**
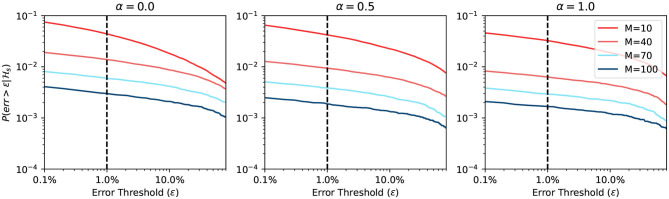
Error probability of static approximation function.

Figure 6 shows the simulation result when using a static approximation function. We can see that the prediction differs when α changes. However, the change is not significant, because the firing rate expectancy roughly equals to *r*_*s*_, no matter what the value of α is. On the other hand, when the BCPNN configuration changes, the probability changes a lot. This change happens because, for large *M*, the expected firing rate is close to 0. Since the expected firing rate approaches 0, it makes it easier for the approximation function to have a correct prediction.

##### 2.4.3.2. Adaptive approximation function

The static approximation function $H_{s}$ is very simple and unable to properly grasp the three different firing rates in the BCPNN. By modifying it, we can easily implement another type of approximation function that factors in the dynamic information available at evaluation time. We call this type of approximation function as adaptive approximation function. It is defined in Equation (10), where r^*^ is the nearest recorded firing rate history and x~U(0,1) is a uniformly distributed random number generated at each prediction. The *a* subscript in H indicates that it is an adaptive function. The $H_{a}$ is very similar to $H_{s}$. The difference between the two functions is that the predicted firing rate here adapts to the true firing rate r^*^.

(10) $$\begin{array}{l} H_{a}:{\mathbb{N}}^{0}\times \mathbb{R}\to 𝔹,\text{so that, }H_{a}:t\mapsto \left(x<r^{*}\right) \end{array}$$

We record the HCU activation status and winning/losing status of each MCU, assuming that spikes come in bursts. The record of such a status can be reasonably infrequent. Additionally, the required information is boolean and thus occupies very little storage space. If we record the winning/losing status of each MCU every 300 simulation steps, only 32 records are needed to cover nearly 10,000 simulation steps. The cost to record such a range for each MCU is equivalent to just a single integer word. Since we assume the HCU and MCU status will not change frequently, we can use the recorded status to determine the real status for any simulation step.

**Figure 7:**
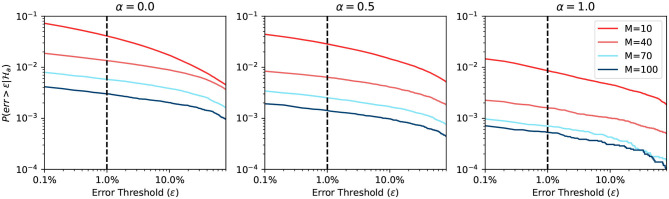
Error probability of adaptive approximation function.

Figure 7 shows the simulation result when using an adaptive approximation function. We can see that the prediction differs more when α changes, compared to the previous static approximation function curve. The explanation is that the adaptive function adapts to the true firing rate. We note here again that for large α, the firing rate has a higher probability of being close to 0. Hence it is easier to predict. When the BCPNN configuration changes, the probability changes a lot. The change follows the same trend of static approximation function simulation.

#### 2.4.4. Summary

**Figure 8:**
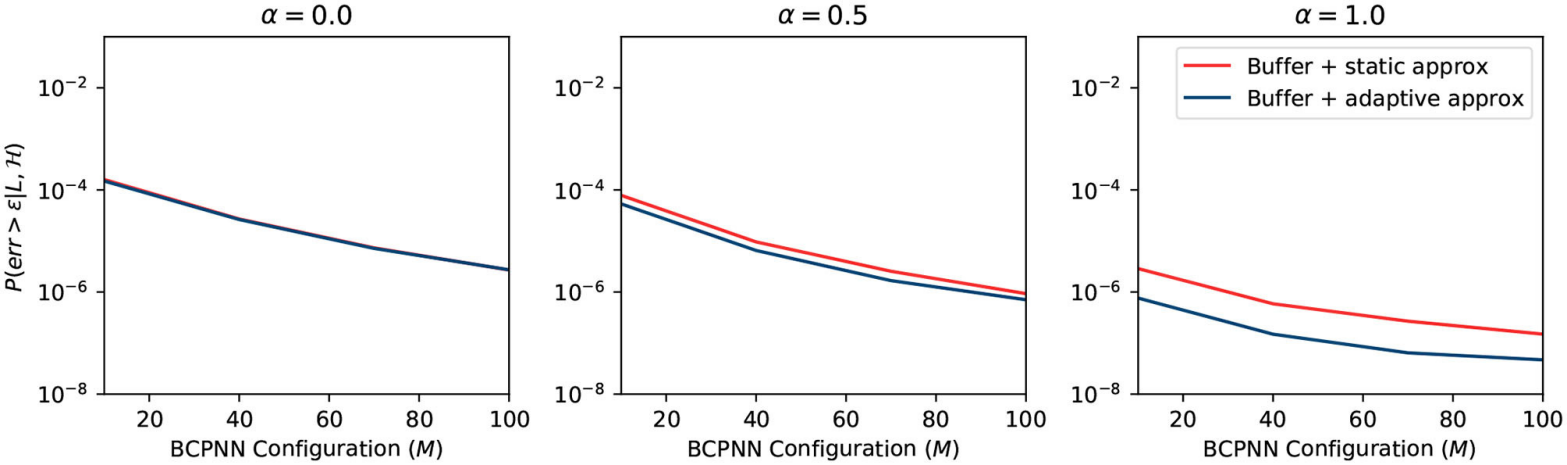
Overall error of different strategy.

We plot the overall error probability in Figure 8. The curve is the multiplication of previous curves in Figures 5–7. It demonstrates two different strategies: (1) buffer + static approximation function and (2) buffer + adaptive approximation function. One can easily tell from the plot that the second strategy dominates the first one. By combining the spike buffer and adaptive approximation functions, we can bound the error as low as to the order of 10^−4^ for any activation level. The worst-case scenario of α = 0 should not happen in any BCPNN simulation. Whereas, for normal BCPNN simulations, α = 1 is usually guaranteed. The *M* is commonly set to 100 when simulating a relatively big network. In such cases, the error bound will be improved dramatically to the order of 10^−8^. Therefore, in normal circumstances, all errors introduced can be considered minor and negligible for the operation of the BCPNN.

The static approximation function leads to a very good error bound as well. Though slightly worse than the adaptive approximation function, it does not require any extra resources. Consequently, it is very efficient to apply the combination of a spike buffer and a static approximation function for most applications. Therefore, we adopt this strategy for the experiments in the results section.

## 3. Experiment Results

In this section, we set up a series of experiments on a GPU platform to examine all the metrics affected by the CUE method. The GPU we use is an Nvidia Quadro K1200 GPU, which can be profiled by the Nvidia GPU profiling tool—*nvprof*. The profiled metrics include storage and performance aspects. We mainly analyze the storage requirements, memory access demand, and memory access efficiency. The approximation function of CUE used in these experiments is the static approximation function due to its simplicity, see section 2.4.4. The experiments are designed to compare the original lazy evaluation method (baseline), the flushing event method, and the CUE method. Both the flushing event method and the CUE method have the same buffer size.

### 3.1. Storage Analysis

In the original lazy evaluation method, we need several variables to be stored in the memory to represent the state of each synapse. This variables are used to track the change of all the traces and time, and they are *p*_*ij*_, *e*_*ij*_, *z*_*i*2_, *z*_*j*2_, *w*_*ij*_, and *t*_*ij*_. Once the column update procedure is removed, all synaptic traces will be updated only when a presynaptic spike *s*_*i*_ occurs. Thus, the timestamp *t*_*ij*_ will be identical to the timestamp *t*_*i*_ in presynaptic traces and can be safely removed. The *z*_*i*2_ is the equivalent *z*_*i*_ trace, and it is stored in the synaptic matrix. In the lazy evaluation method, *z*_*i*_ and *z*_*i*2_ could be updated at different times, so we have to make a copy (*z*_*i*2_) inside the synaptic matrix. After applying CUE, *z*_*i*2_ will only be updated when an *s*_*i*_ occurs, its value should be identical to *z*_*i*_ for all synapses corresponding to the same presynaptic trace. We can now safely remove the *z*_*i*2_ trace.

The flushing event method still keeps the column update. The storage of the flushing event method thus is identical to the lazy evaluation method except that it requires some extra buffer storage for post-synaptic spikes. Overall, the flushing event method doesn't change the storage requirement.

**Figure 9:**
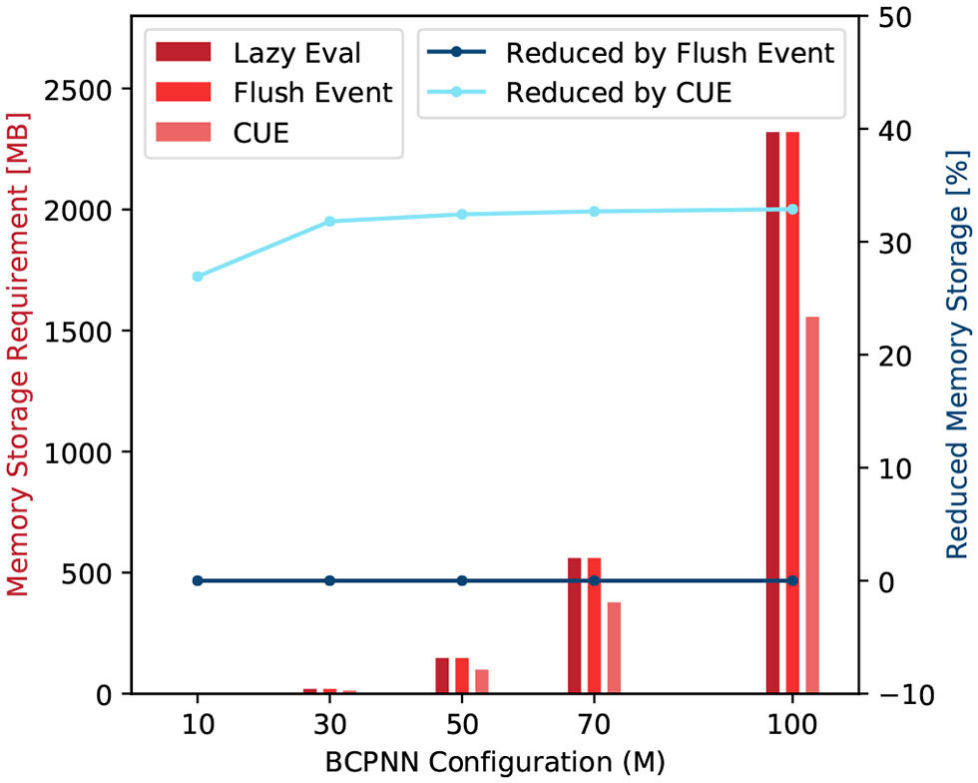
Memory storage requirement comparison (red) and requirement reduction by the flushing event method and the CUE method (blue).

Note that both *t*_*ij*_ and *z*_*i*2_ traces are synaptic variables that dominate the storage cost for BCPNN simulation because the amount of data representing synaptic traces is proportional to the product of the number of pre- and post-synaptic units. Figure 9 summarize the storage comparison of the original lazy evaluation method and CUE method. The figure shows that we save around 33% of memory by applying CUE. The 33% of reduction is due to the elimination of *t*_*ij*_ and *z*_*i*2_, which are two out of six synaptic traces.

### 3.2. Performance Analysis

In this section, we analyze the performance of each method in terms of the memory access demand, the memory access efficiency, and the latency of the row/column update CUDA kernels. The first two factors focus on the memory aspects but neither of them can characterize the performance alone. The overall performance is instead characterized by the latency of the row/column update CUDA kernels. In our experiments, we implement and test a series of small BCPNNs with configuration *M* × *M*, where *M* ∈ {10, 30, 50, 70, 100}. Each of these networks is trained to remember 10 patterns, where each pattern is trained for 500 ms.

#### 3.2.1. Memory Access Demand

Compared with the original lazy evaluation method, the memory access demand requested by the column update is eliminated. We remind the reader that, in the original lazy evaluation method, the column update roughly demands the same amount of memory access as a row update. However, when applying CUE, the memory access demand of a row update increases slightly due to the access for spike buffer. Therefore, the improvement in memory demand should be <50%.

We record two global memory access related metrics: *global memory load transactions* (*N*_*ld*_) and *global memory store transactions* (*N*_*st*_). They represent the global memory access demand requested by the CUDA kernels. The Equation (11) calculates the memory demand of those row/column update kernels.

(11) $$\begin{array}{l} D=k_{L1}*\left(N_{ld}+N_{st}\right) \end{array}$$

**Figure 10:**
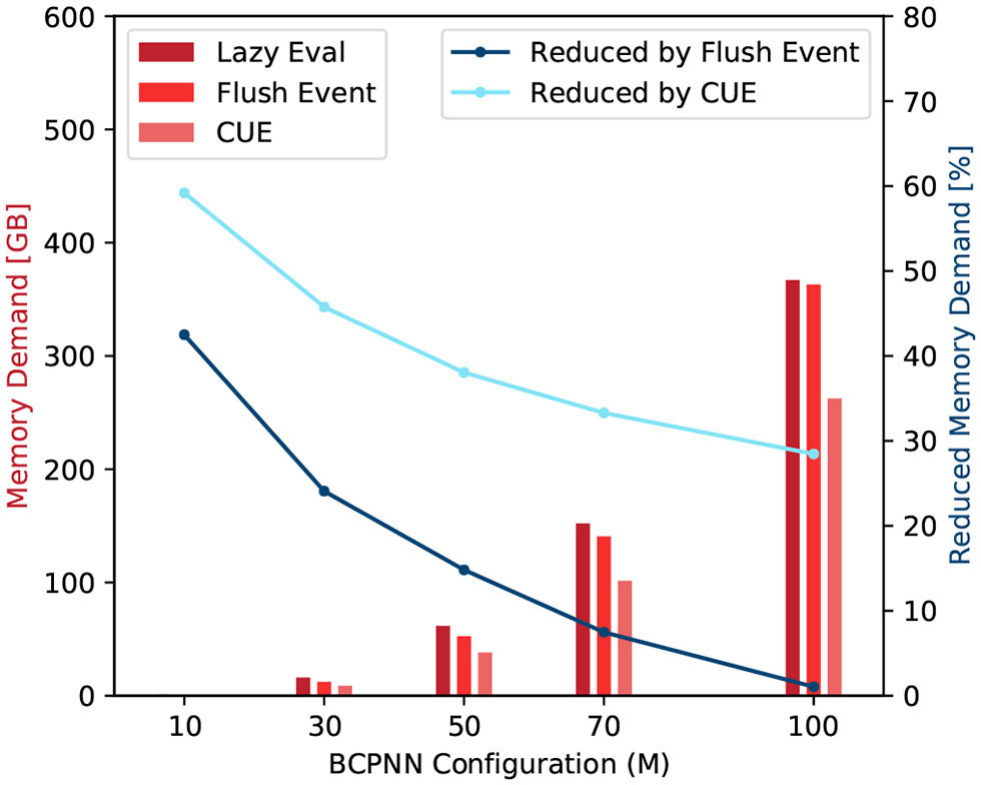
Memory access demand comparison (red) and memory demand reduction by the flushing event method and the CUE method (blue).

The scaling factor *k*_*L*1_ is set to 128 because the size of the Quadro K1200 GPU's L1 cache line is 128 Bytes. The 𝔻 represents the amount of data in Bytes that is needed to be loaded from, or stored to, the global memory. Figure 10 shows the comparison among the original lazy evaluation method, the flushing event method, and the CUE method. From the figure, we can see that half of the memory access demand of a small network is eliminated by CUE method due to the elimination of the column update process. In a bigger network, the memory demand is improved by one third. The flushing event method however reduces the memory access demand in a much smaller proportion. It can reduce the invocation of column update, and save memory access for active rows. However, the access of the spike buffer is a non-negligible overhead. Overall, the flushing event method saves memory access demand. However, for big networks, the saving is largely canceled by the overhead of accessing the spike buffer.

#### 3.2.2. Memory Access Efficiency

In this experiment, we profile the following four metrics: *DRAM read transactions* (*N*_*rd*_), *DRAM write transactions* (*N*_*wr*_), *global memory load transactions* (*N*_*ld*_), and *global memory store transactions* (*N*_*st*_). The *N*_*rd*_ and *N*_*wr*_ are the recorded READ and WRITE operation count from/to the DRAM. They represent the actual DRAM access. The Equation (12) defines a parameter called DRAM access rate γ.

(12) $$\begin{array}{l} \gamma =\frac{k_{L2}\left(N_{rd}+N_{wr}\right)}{k_{L1}\left(N_{ld}+N_{st}\right)}\times 100\% \end{array}$$

Here, the *k*_*L*2_ and *k*_*L*1_ are the L2 and L1 cache line size, respectively.

**Figure 11:**
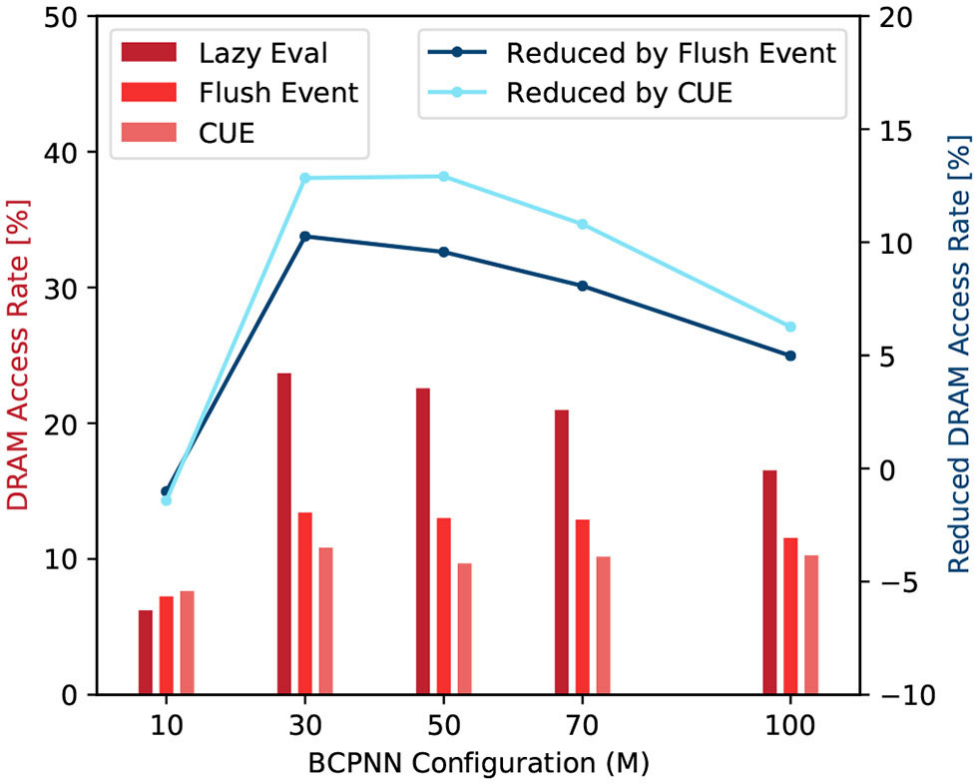
DRAM access rate comparison (red) and DRAM access rate reduction by the flushing event method and the CUE method (blue).

From Equation (12), we can see that a large γ value means a large number of real DRAM access and a high cache-miss rate. Figure 11 shows the DRAM access rate of several BCPNN configurations. We can see that the DRAM access rate is reduced by the CUE method, although the reduction is less dramatic for bigger networks. This is because the column-wise memory access demand has already been eliminated; the improvement has been shown in Figure 10. The improvement that is shown in Figure 11 only presents the average memory access efficiency using the CUE method, compared to the lazy evaluation method.

We can also observe that the flushing event method improves memory access efficiency compared to the lazy evaluation method. It is because even though the memory access demand for the spike buffer is equivalent to the memory access demand of a single cell column update, the memory access for the buffer is coalesced, thus much more efficient than the column update. When the flushing event happens, it also updates according to the spike buffer and resets the spike buffer to reduce the invocation of the column update kernel. However, the flushing event method is not as good as the CUE method because it still keeps the extremely inefficient column update.

If we look at the trend of the DRAM access rate, we can see that it behaves abnormally when *M* = 10. That is because the network is so small, the L2 cache can hold almost the entire network. Therefore, the lazy evaluation method is efficient. For *M* ≥ 30, the column update becomes slightly more efficient with the increase of network size. It's mainly due to the variation of the proportion of empty rows in the synaptic matrix that affects the efficiency of the column update. We don't explain the complete causality of this phenomena since it's not very related to our memory access efficiency comparison and it requires a lot of implementation details related to the GPU platform.

#### 3.2.3. Latency

**Figure 12:**
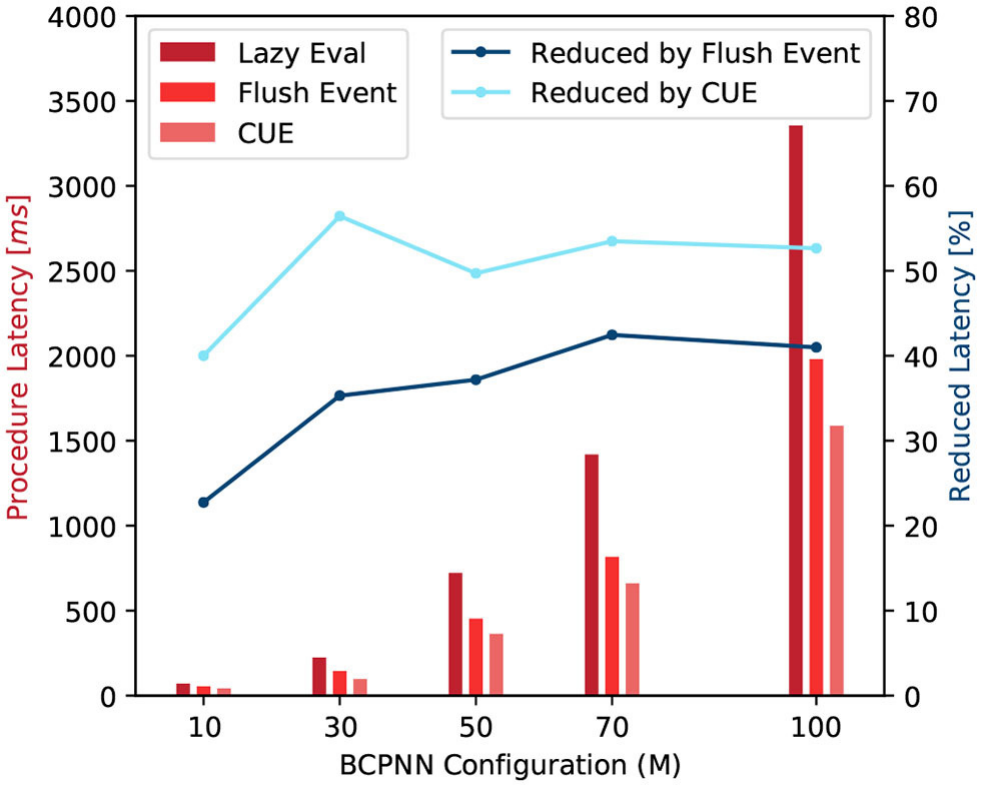
Latency comparison of synaptic matrix update procedures (red) and latency reduction by flushing event method and CUE method (blue).

With the improvement in terms of memory demand and memory access efficiency, the overall latency of function call is reduced. We test the average latency of a row and column update procedures. Figure 12 shows that compared to the original lazy evaluation method, which requires both row and column update, our CUE strategy reduces the latency of the synaptic matrix update. The average latency is reduced by about 50%. The reduction in latency is mainly due to memory optimization and the elimination of issuing the column update kernel, which has some overhead.

The flushing event method also improves the overall performance compared to the lazy evaluation method. However, due to the column update process, the improvement is less than the CUE method which eliminates the entire column update.

For both blue lines in Figure 12, when *M* = 50, the reduction drops a little. It is because that the column update efficiency changes with the change of network size. But the change rate is not very uniform due to the nature of the GPU platform. It will be more efficient if the network configuration makes the memory access pattern to fit the GPU warp size. *M* = 50 is one of such configuration points.

## 4. Discussion

In this paper, we have discussed the BCPNN memory access problem introduced by the lazy evaluation method. An algorithmic optimization has been proposed to tackle this issue. The proposed Column Update Elimination (CUE) method eliminates the column update and merges it with the row update, with the help of spike history buffer and approximation function. Using the CUE method, we gain not only memory access efficiency but also other improvements, such as the reduction of memory storage, memory access demand, etc. We also show that our algorithmic modification only introduces negligible errors and does not compromise the functionality of the BCPNN.

In this section, we further examine the potential of the proposed method. We focus on the new possibilities after the column update has been eliminated, and other learning rules that CUE method can fit in. Finally, based on what we have achieved, we describe an outlook that could improve BCPNN simulation even further.

### 4.1. Exploiting the Temporal Locality of Spike Train

Memory access efficiency can be further improved by architectural optimization. We have analyzed the pattern of spike train in section 2.4.1. Spikes are generated in burst mode regulated by stimulus pattern. Usually, the change of these stimulus patterns is infrequent. Thus, when the neural network is in the middle of a stable stimulus pattern, the firing patterns of spikes are also stable.

We have analyzed the percentage of winning MCUs in both active and silent HCUs. Even in completely active *H* × *M* BCPNN (α = 1), the fraction of winning MCUs which fire frequently is just $\frac{1}{M}$. Therefore, only a fraction of $\frac{1}{M}$ connections will be active at each simulation step, assuming uniform interconnections. Therefore, when the firing patterns are stable, the part of synaptic traces that need to be frequently updated is also stable, and the global memory access patterns of synaptic traces are stable as well.

In this paper, we have eliminated the non-coalesced column-wise memory access pattern. With stable global memory access patterns, we can cache the frequently updated fraction of synaptic traces by designing a large enough cache between the memory and computation unit. The single global memory access pattern guarantees that the cache system will not be interrupted by other memory access patterns.

Unfortunately, the estimated size of such a cache usually is much bigger than any off-the-shelf commercial computer architecture. The insufficient size of the cache will lead to frequent swapping data between the cache and DRAM, thus significantly compromises the memory access efficiency. The customization of the cache system becomes necessary and can only be done in custom hardware architecture, such as ASICs.

### 4.2. CUE Method for STDP

The STDP learning rule changes the synaptic weights based on the correlation of pre- and post-synaptic spikes. The amount of change depends on the time difference between the pre- and post-synaptic spikes. A pre- and post-synaptic spike pair for a synaptic connection 〈*s*_*i*_, *s*_*j*_〉 occurs at time 〈*t*_*i*_, *t*_*j*_〉. If *t*_*i*_ < *t*_*j*_, they are correlated. Otherwise, they are anti-related. One commonly used method to calculate such causal strength is described in Equation (13).

(13) $$\begin{array}{l} \Delta w_{ij}=\{\begin{array}{c} +A_{+}\cdot e^{-\frac{t_{j}-t_{i}}{\tau }},\text{if }t_{i}<t_{j}\\ -A_{-}\cdot e^{-\frac{t_{i}-t_{j}}{\tau }},\text{if }t_{i}>t_{j} \end{array} \end{array}$$

We can see that the update of weights in the STDP learning rule is triggered by pre- and post-synaptic spikes, just like the BCPNN learning rule. It is natural to organize the synaptic weights of STDP learning as a matrix stored in the memory. Because of its triggering mechanism and data structure, the STDP learning rule will suffer from the same dual memory access pattern issue as a lazy evaluation method.

By proposing the same solution to STDP learning, we can use a post-synaptic buffer and an approximation function to delay the update of the post-synaptic triggered column update. The buffer size *L*, and the type of approximation function H will be different for STDP learning. Further analysis and simulation should be done to investigate the error bound. For example, the probability density function (PDF) of the firing rate in STDP might be different. It might be enough for STDP by just using the spike history buffer. However, the principle of optimizing memory access efficiency remains the same for both STDP and BCPNN learning rules.

### 4.3. Outlook

In the future, we could also explore the option of hardware architecture to improve the BCPNN simulation further. For example, we could dimension a big enough cache that can hold the complete stationary synaptic traces to avoid DRAM memory access. We could also use non-von Neuman architecture, such as memristor, which could potentially avoid the memory access problem. Other algorithmic modifications combined with approximate computing, such as delayed stochastic row update, could also improve the overall BCPNN simulation.

## Data Availability Statement

The raw data supporting the conclusions of this article will be made available by the authors, without undue reservation.

## Author Contributions

The initial idea proposed in the paper came from AL and AH. YY further developed the method by introducing the approximation function, proposed the methodology for error analysis, and performed the experiments to verify the proposed method. DS and RJ helped with the refinement of method especially the error analysis part. All authors contributed to the article and approved the submitted version.

## Conflict of Interest

The authors declare that the research was conducted in the absence of any commercial or financial relationships that could be construed as a potential conflict of interest.
